# Expression of activin A in liver tissue and the outcome of patients with biliary atresia

**DOI:** 10.3389/fped.2024.1457837

**Published:** 2024-11-15

**Authors:** Petra Džepina, Marijana Ćorić, Matea Kovačić Perica, Mirna Natalija Aničić, Ruža Grizelj, Jurica Vuković

**Affiliations:** ^1^ Department of Pediatrics, School of Medicine, University Hospital Centre Zagreb, University of Zagreb, Zagreb, Croatia; ^2^ Department of Pathology and Cytology, School of Medicine, University Hospital Centre Zagreb, University of Zagreb, Zagreb, Croatia; ^3^ School of Medicine, University of Zagreb, Zagreb, Croatia

**Keywords:** biliary atresia, activin A, hepatoportoenterostomy, liver immunohistochemistry, survival with native liver

## Abstract

Biliary atresia (BA) is a rare disease of unknown etiology which leads to cirrhosis and death if left untreated. The standard of care is an early hepatoportoenterostomy (HPE). Long-term follow-up is mandatory, during which most patients will require a liver transplant. Activin A belongs to the transforming growth factor-β (TGF-β) superfamily. TGF-β is a central regulator in chronic liver disease. We have studied the expression of activin A in liver tissue collected intraoperatively during the HPE. We included patients who underwent HPE in a single medical center. Clinical, ultrasonographic, and pathohistological data were collected. Activin A immunostaining was performed. Expression in the bile duct epithelium and hepatocytes was scored as either weakly positive, moderately positive, or strongly positive. Patients were then divided into three groups accordingly. We observed the outcome after the HPE at 3 months, 2 years, and at the end of follow-up. The study encompassed 37 patients. At 3 months after HPE, 92.3% of those with a weakly positive activin A reaction (group A) achieved good jaundice clearance, whereas only 44.4% of those with a moderately (group B) and 40% of those with a strongly positive reaction (group C) achieved good jaundice clearance (*p* = 0.008). Furthermore, 2 years after the HPE, 92.3% of those in group A survived with native liver (SNL), but only 33.3% of those in group B and 46.7% of those in group C had SNL (*p* = 0.007). At the end of follow-up, 83.3% of those in group A survived with native liver, as did 33.3% in group B and 40% in group C. Activin A is a valuable pathohistological predictor of the outcome of BA after an HPE.

## 1 Introduction

Biliary atresia (BA) is a rare disease of unknown etiology that presents in newborn infants. It is characterized by inflammatory obliterative cholangiopathy, affecting both the intrahepatic and extrahepatic bile ducts (1–7). BA leads to the development of cirrhosis, liver failure, and death within the first 2 years of life if left untreated (1–7). The incidence of BA depends on the geographical location and is most common in Asian countries (1:5,000 live births in Taiwan), whereas in Europe the incidence is lower (1:19,000 live births in the Netherlands) (1–3). BA occurs slightly more often in females than in males (1.25:1) (1). The diagnosis is established according to the clinical and ultrasound findings and the exclusion of other causes of conjugated hyperbilirubinemia. In most cases, it is still necessary to confirm the diagnosis with an intraoperative cholangiogram (1–3). At the time of diagnosis, the extrahepatic bile ducts are completely obliterated, whereas the intrahepatic bile ducts show signs of inflammation and fibrosis, with the accumulation of bile and variable amounts of multinuclear hepatocytes surrounding the ducts (1–3). Thus far, the only successful treatment of biliary atresia is a surgical hepatoportoenterostomy (HPE), which enables the drainage of bile from the remaining bile duct of the porta hepatis into the small intestine (1–3, 8, 9). The complications resulting from an HPE are numerous (e.g., frequent postoperative cholangitis and incomplete drainage of bile with consequent progression of cholestasis), and the disease progresses to cirrhosis of the liver in many patients with the subsequent need for transplantation even after HPE (8, 9). Biliary atresia is also the most common indication for liver transplantation in children (1–3, 8, 9).

BA can be divided into at least two clinical types: fetal and perinatal. In the fetal form, newborns develop conjugated hyperbilirubinemia and acholic stools within the first week of life, and there is no jaundice-free period. In the perinatal form, jaundice and acholic stools develop after a certain jaundice-free period (10).

Furthermore, there are more distinguishable entities such as biliary atresia splenic malformation (BASM) or cat-eye syndrome, along with cystic BA. This diversity suggests etiological and probably pathogenetic heterogeneity (11). Various causes have been proposed thus far (e.g., exposure to viral infections, genetic predisposition, or response to the bile duct epithelium's own antigens) (1, 10–18). There is also a distinct BA variant in Egyptians, which seems to be caused by an interaction between congenital aflatoxicosis in neonates and the glutathione S-transferase M1 null genotype (19). The immune system plays an important role in the development of this disease, as shown by the presence of inflammatory cells and various adhesion molecules in the bile duct area (15). There is a lingering question of whether liver histopathology can additionally help in the prediction of BA treatment outcomes (10–13).

Activin A is a member of the family of transforming growth factors (TGF-β) (20–28). It is found in various tissues and is secreted by macrophages, Th2 lymphocytes, and hepatocytes. Recent research has shown that activin A causes the inhibition of hepatocyte growth, suppresses liver regeneration, and induces hepatocyte apoptosis (20–28). Excessive secretion of activin A in the liver has thus far been detected in liver fibrosis, liver cancer, and viral hepatitis (23–28).

## 2 Methods

### 2.1 Study design

The medical records of patients who underwent an HPE at the Department of Pediatrics, Division of Gastroenterology, Hepatology and Nutrition, University Hospital Centre Zagreb, from 1986 to 2019 were reviewed retrospectively. The diagnosis of BA was established according to standard clinical, biochemical, nuclear, ultrasonographic, histologic, and operative findings. Clinical findings included conjugated hyperbilirubinemia in biochemistry analysis, history of acholic stools, dark urine, and hepatomegaly. The diagnosis was confirmed with hepatobiliary scintigraphy and a liver biopsy. We set 30 June 2021 as the end of follow-up.

### 2.2 Inclusion criteria

**Figure 1:**
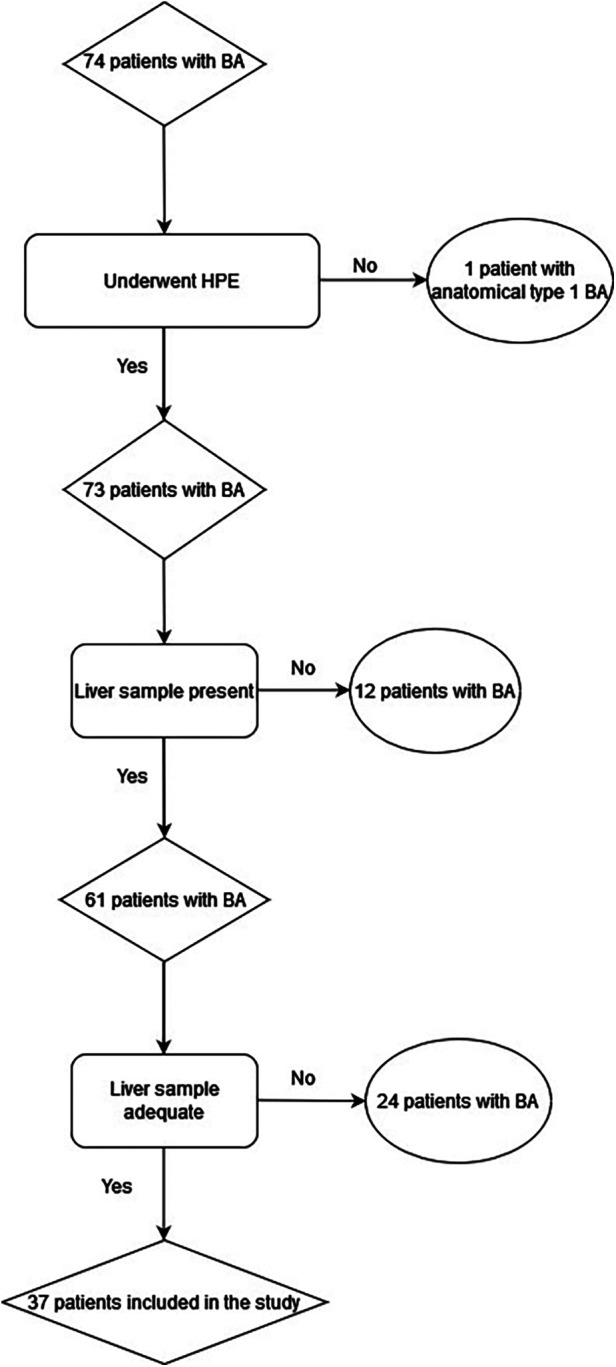
Flow chart of the sample size methodology.

We included all children with BA who had detailed clinical information, at least 2 years of follow-up after the HPE, and wedge liver biopsy specimens in good condition in paraffin blocks (Figure 1).

### 2.3 Exclusion criteria

We excluded any patients with biliary atresia who underwent surgical treatment other than an HPE.

### 2.4 Data gathering

Data on the patient’s date of birth, birth weight, sex, the date of onset of jaundice with conjugated hyperbilirubinemia and acholic stools, presence of congenital extrahepatic anomalies, the date of the HPE, and liver histology results from the biopsy obtained during the HPE were gathered, as well as the age of onset of liver cirrhosis. Diagnostic histologic findings for biliary obstruction included ductular proliferation, cellular and canalicular bile stasis, and portal edema and fibrosis. Furthermore, we gathered data including months of survival with native liver (SNL) after the HPE, the date of either liver transplantation or death, and the date of the last recorded out-patient visit for drop-outs.

### 2.5 Immunohistochemistry

A liver wedge biopsy was taken in all patients at the time of HPE, at the porta hepatis. Specimens were fixed in 10% formalin and then embedded in paraffin after processing the tissue. In all the biopsies, conventional stains (hematoxylin and eosin, Mallory, Gomory) were performed on 3- to 4-μm-thick paraffin sections. Hepatic fibrosis was graded on the basis of the Ishak classification: grade 0, no fibrosis; grade 1, fibrous expansion of some portal areas; grade 2, fibrous expansion of most portal areas; grade 3, fibrous expansion of most portal areas with occasional portal to portal bridging; grade 4, fibrous expansion of portal areas with marked bridging (portal to portal and portal to central); grade 5, marked bridging (portal to portal and/or portal to central) with occasional nodules–incomplete cirrhosis; and grade 6, cirrhosis (29). On all the biopsy samples, we performed antigen retrieval in a Dako PT Link device (Dako, Agilent Technologies, Santa Clara, CA, USA) in the appropriate buffer [Envision Flex Target retrieval solution LOW (Dako, Agilent Technologies, Santa Clara, CA, USA)]. Afterward, the samples were dyed in a Dako TechMate device (Dako, Agilent Technologies, Santa Clara, CA, USA) using the standard dyeing process. Purified rabbit polyclonal primary activin A antibody was applied to the samples at a concentration of 1:100 (RRID: AB_2125870, NBP1-30928, Novus Biologicals, Colorado, USA). Given our study duration and the viability of our paraffin-embedded samples, we chose a polyclonal antibody for the immunohistochemical (IHC) staining as it increases the chance of detecting epitopes that are still available in fixed samples with a low protein quantity (30, 31). For visualization, the EnVision FLEX kit was used (Dako, Agilent Technologies, Santa Clara, CA, USA). Finally, the specimens were contrast stained with hematoxylin and dehydrated in solutions as follows: 96% ethyl alcohol 2 × 5 min, 100% ethyl alcohol 2 × 5 min, and 100% xylene 2 × 5 min.

**Figure 2:**
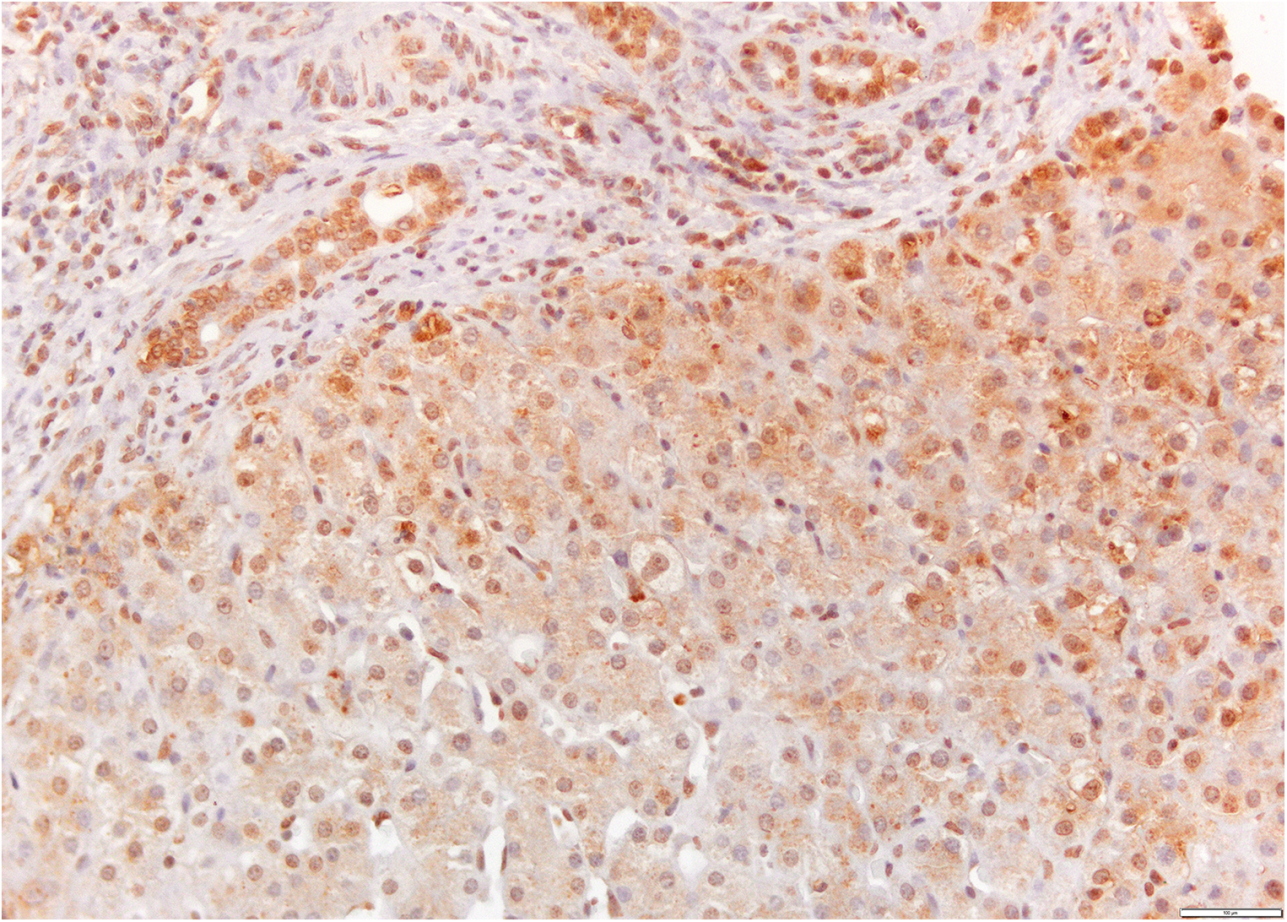
Activin A immunostaining of a liver sample from a patient with biliary atresia. The expression is present in hepatocytes and cholangiocytes in the portal space (brown-colored, ×20 magnification).

The samples were analyzed with a light microscope, in at least five fields of view under high magnification (×400) by one pathologist who was unaware of patient outcomes (Figure 2). The activin A expression was established using our own semiquantitative method, according to the following principle: no staining in the hepatocyte cells and biliary epithelial cells (BECs) (−); up to 10% of the hepatocytes and biliary epithelial cells have a positive reaction, weakly positive reaction (+); from 10% to 50% of hepatocytes and biliary epithelial cells positive, moderately positive reaction (++); more than 50% of hepatocytes and biliary epithelial cells positive, strongly positive reaction (+++).

### 2.6 Outcomes

We observed the patient outcomes at 3 months after the HPE, 2 years after the HPE, and at the study end point. The patients were divided into three groups based on their outcomes at 3 months as indicated by stool color and total bilirubin (TB). An excellent outcome was represented by the presence of colored stools and TB lower than 30 µmol/L. A good outcome was represented by the presence of colored stools and TB higher than 30 µmol/L and lower than 90 µmol/L, whereas a bad outcome was represented by the presence of either acholic stools or persistent jaundice. The outcome at 2 years after the HPE was divided into SNL or any other outcome (liver transplantation and/or death, study drop-out). Liver cirrhosis was defined when any sign of portal hypertension (inverse portal blood flow, ascites, esophageal varices, hypersplenism, or splenomegaly) was noted. The outcome at the study end point was observed as SNL, liver transplantation (LTx), or death.

## 3 Statistical analysis

The data analyses were performed using R software (version 4.1.2). Categorical data were descriptively presented as absolute and relative frequencies, and numerical data, depending on the distribution, as means and standard deviations or medians and interquartile ranges. The Kolmogorov–Smirnov test was used to test the normality of the distributions. Fisher's exact test was used to test differences between categorical variables, and a one-way ANOVA and the Kruskal–Wallis test for differences between numerical variables. In addition, survival estimates with native liver were based on the Kaplan–Meier method and log-rank test. *P*-values <0.05 were considered statistically significant.

## 4 Results

### 4.1 Demographics and subgroup division

In total, 37 patients met the inclusion criteria. Nine of them were included in an earlier report summarizing the incidence and outcomes of patients with BA (32). Demographic data are presented in Tables 1, 2. Within our cohort, 13 patients had a weakly positive reaction in the activin A immunostaining (+)—group A, 9 had a moderately positive reaction (++)—group B, and 15 had a strongly positive reaction (+++)—group C.

**Table 1:** Clinical characteristics of the study group.

Sex (%)	Male	18 (48.6)
	Female	19 (51.4)
Malformations present (%)	Yes	8 (21.62)
	No	29 (78.38)
Median age at the HPE, days (range)		76 (22–192)
Median follow-up after the HPE, years (range)		6.2 (0.6–23.8)
LTx during the follow-up (%)	Yes	12 (33.3)
	No	24 (66.6)

**Table 2:** Associated anomalies^a^.

Anomaly	Total, *N* (%)
BASM	3 (8.1)
Gastrointestinal	4 (10.8)^b^
Cardiovascular	3 (8.1)^b^
Urogenital	1 (2.7)
Musculoskeletal	1 (2.7)

^a^ Twelve anomalies in eight patients.

^b^ BASM overlap not included.

### 4.2 Malformations present

There was one BASM patient in every group. There were only two patients with a single anomaly (one with ectopic pancreas and the other with pyeloureteral stenosis). Besides these two which were not readily recognized from established BA registries, the other anomalies were those usually encountered (gastroschisis, ventricular septal defect, finger malformations, atrial septal defect, and hypoganglionosis of the colon).

### 4.3 Outcomes

Table 3 presents preoperative, pathohistological, and analyzed outcomes for each group separately. At 3 months after the HPE, more than two-thirds of the patients in group A (69.2%) achieved excellent jaundice clearance. The same outcome was noted in 11.1% of the patients in group B, and 33.3% of the patients in group C, respectively (*p* = 0.007). Likewise, 2 years after the HPE, 12 out of the 13 patients (92.3%) from group A had survived with their native liver, as had 33.3% of the patients in group B and 46.7% in group C (*p* = 0.007). At the end of the study, 10 out of the 12 patients in group A (83.3%), 3 out of the 9 patients in group B (33.3%), and 6 out of the 15 (40%) in group C survived with native liver. The log-rank test did not show a significant difference in the SNL estimate between the three groups given death and transplantation as the main events, respectively (*p* = 0.136 and *p* = 0.071). There was no significant difference in the median age at which the HPE was performed across the three groups of patients (*p* = 0.810).

**Table 3:** Comparison of the characteristics and outcomes of infants according to the activin A expression in the liver wedge biopsy.

		Group A	Group B	Group C	*p*-value^a^
*n* (%)		13 (35.1)	9 (24.3)	15 (40.6)	
Sex (%)	Female	8 (61.5)	6 (66.7)	5 (33.3)	0.239
	Male	5 (38.5)	3 (33.3)	10 (66.7)	
Anomalies (%)	No	12 (92.3)	7 (77.8)	10 (66.7)	0.307
	Yes	1 (7.7)	2 (22.2)	5 (33.3)	
Age at HPE in days [median (IQR)]		82.00 (64.00–86.00)	76.00 (39.00–106.00)	70.00 (54.00–84.50)	0.810
BA type (%)	Fetal	2 (15.4)	4 (44.4)	4 (26.7)	0.333
	Perinatal	11 (84.6)	5 (55.6)	11 (73.3)	
Ishak index [mode (range)]		4 (1–6)	5 (1–6)	6 (1–6)	0.754
Ishak index	1–4	7 (58.3)	4 (44.4)	7 (46.7)	0.835
	5–6^b^	5 (41.7)	5 (55.6)	8 (53.3)	
Age at liver cirrhosis in months [median (IQR)]		24.00 (12.25–41.75)	7.00 (7.00–15.50)	8.00 (6.75–9.00)	0.042
Number of cholangitis attacks (%)	No attacks	12 (92.3)	5 (55.6)	10 (66.7)	0.304
	1 attack	1 (7.7)	2 (22.2)	3 (20.0)	
	>1 attack	0 (0.0)	2 (22.2)	2 (13.3)	
Outcome at 3 months after HPE (%)	TB <30 µmol/L and colored stools	9 (69.2)	1 (11.1)	5 (33.3)	0.007
	TB 30–90 µmol/L and colored stools	3 (23.1)	3 (33.3)	1 (6.7)	
	TB >90 µmol/L and/or acholic stools	1 (7.7)	5 (55.6)	9 (60.0)	
Outcome 2 years after HPE (SNL, death or Tx) (%)	SNL	12 (92.3)	3 (33.3)	7 (46.7)	0.007
	Other (death or Tx)	1 (7.7)	6 (66.7)	8 (53.3)	

*n*, number; HPE, hepatoportoenterostomy; IQR, interquartile range; BA, biliary atresia; TB, total bilirubin; SNL, survival with native liver; Tx, transplantation.

Values are numbers for nominal variables and median (range) for continuous variables. The characteristics were compared across groups using ANOVA and the Kruskal–Wallis test for continuous variables and Fisher's exact test for categorical variables.

^a^ The *p*-values in parentheses correspond to comparison between group A, group B, and group C.

^b^ Ishak fibrotic stages 5 and 6 represent incomplete and complete cirrhosis.

### 4.4 Liver cirrhosis comparison

The patients in groups B and C developed signs of liver cirrhosis sooner (median age in months 7.0, IQR 7–15.5; and 8.0, IQR 6.75–9.00, respectively) than the patients in group A (median age in months 24.00, IQR 12.25–41.75) (*p* = 0.042). The mode of the Ishak index across three groups was somewhat similar [4 (1–6), 5 (1–6), and 6 (1–6), *p* = 0.754]. However, there was no significant difference between the Ishak index score of liver fibrosis and activin A expression in the liver wedge biopsies across the three patient groups (*p* = 0.835).

## 5 Discussion

**Figure 3:**
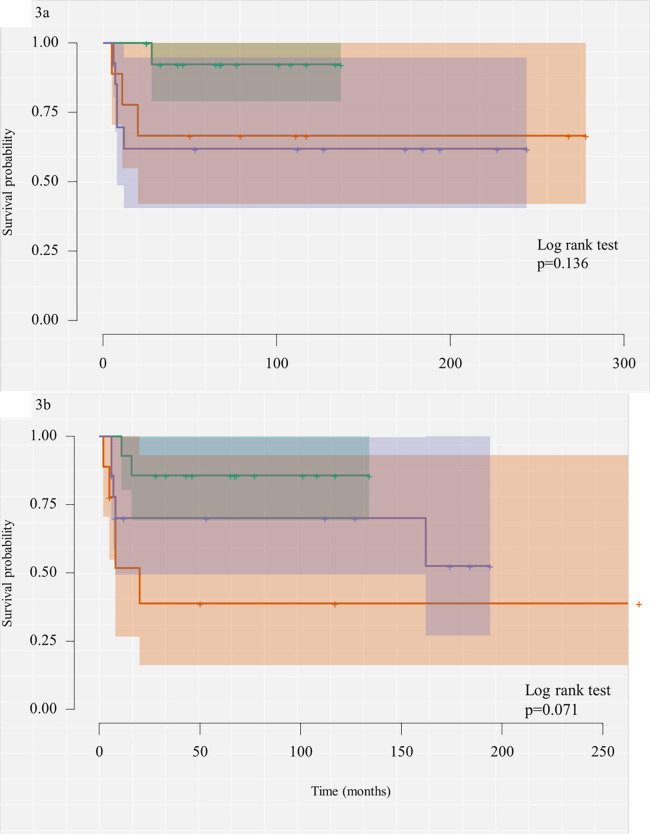
**(a)** Kaplan–Meier curve of the survival analysis estimating SNL probability with death as the main event. **(b)** Kaplan–Meier curve of the survival analysis estimating SNL probability with liver transplantation as the main event.

The main finding of our study is that the patients with a moderate (group B) or strong expression of activin A (group C) on the liver wedge biopsy had worse early outcomes than those with weakly expressed activin A (group A). The patients with moderately or strongly expressed activin A also developed liver cirrhosis sooner than patients with a weak expression of activin A. Although the log-rank test did not show a significant difference in the SNL estimate across the three groups of patients, the Kaplan–Meier curves clearly show a difference with better SNL in patients with weakly expressed activin A (Figures 3a,b). An obvious concern in our study, which included only 37 patients, is the possibility that the statistical power for determining differences between these three groups was limited.

One of the known predictors of biliary atresia outcome is the age at which the HPE is performed (2, 33–35), however, in our cohort, there were no significant differences across the three groups in the median age at which the HPE was executed. Neither the type of BA nor the associated anomalies showed notable differences across the three groups of patients. Some of the variables that were previously shown to influence the outcome of BA were not part of our study design and that may be a limitation for interpreting the role of activin A as a predictor of outcome in our patients.

Activin A has been shown to inhibit mitogen-induced DNA synthesis and induce apoptosis in hepatocytes *in vitro* and *in vivo* (20). Moreover, it inhibits the proliferation and induces apoptosis of hepatocytes, contributing to the termination of liver regeneration. In rat models of liver fibrosis and cirrhosis, activin A expression is increased, and plasma activin A levels are elevated in patients suffering from acute liver failure, hepatitis, alcohol-induced cirrhosis, hepatocellular carcinoma, non-alcoholic fatty liver disease (NAFLD), and non-alcoholic steatohepatitis (NASH). Activin A also regulates the restoration of liver architecture after a partial hepatectomy by stimulating collagen production in hepatic stellate cells (HSC) and tubulogenesis of sinusoidal endothelial cells. Stimulation of collagen production may also contribute to liver fibrosis (26–28, 35–37). The reports on activin A expression in biliary epithelium are conflicting as there are reports of activin A-induced inhibition of DNA synthesis in BECs and reports of increased IHC staining of BEC in liver disease (38, 39). Since both ductular proliferation and ductopenia can be present in BA, we considered activin A staining of the biliary epithelium to be equally as important as the staining of hepatocytes (40). To the best of our knowledge, there are no studies on activin A expression in the livers of patients with BA, and there is no IHC scoring system for activin A in human liver tissue, which are the main restrictions in our study. Despite a considerable amount of data in immunohistochemistry, there is still a lack of standardization, especially in the post-analytical stage, which makes comparisons of the results of different studies difficult or impossible. As we were not able to find an already established scoring system, we devised our own. We only used the number of cells stained with activin A, and given the antibody polyclonality, we did not measure the color intensity to be more objective and to nullify the background staining. This approach has been used before in other tissues (41). There are significant connections between activin A expression and fibrotic changes in liver tissue from a liver wedge biopsy (25, 27). Our study did not show a significant connection between the Ishak classification of liver fibrosis and activin A expression. However, there was no significant difference between the three groups of patients based on the mean Ishak index at the time of the liver wedge biopsy, which showed significant fibrotic liver changes. It has been shown there is a significant effect of the presence of neutrophils in the liver tissue on the pathogenesis and outcome of patients with BA, especially the CD4+/CD8+ ratio (42). Even so, the question of the extent of the effect of liver fibrosis on the outcome of patients with BA remains. Therefore, it may be plausible that activin A expression in otherwise histologically indistinguishable tissues can serve as a more sensitive marker of fibrosis and be a part of future, more accurate assessment tools for the prediction of SNL in patients with BA.

## 6 Conclusion

Our results suggest that activin A expression in the liver of patients with BA before treatment might be a valuable additional tool in the complex search for more accurate predictors of outcomes after an HPE.

## Data Availability

The raw data supporting the conclusions of this article will be made available by the authors, without undue reservation.
